# Treatment of acute aortic dissection type A with paraplegia and distal limb ischemia within a hybrid operating room

**DOI:** 10.1186/s40792-022-01505-7

**Published:** 2022-08-02

**Authors:** Venny Lise Kvalheim, Maria Devold Soknes, Guttorm Lysvold Jenssen, Rune Haaverstad

**Affiliations:** 1 Section of Cardiothoracic Surgery, Dept. of Heart Disease, Haukeland University Hospital, Bergen, Norway; 2 Dept. of Clinical Science, Faculty of Medicine, University of Bergen, Bergen, Norway; 3 Dept. of Radiology, Haukeland University Hospital, Bergen, Norway

**Keywords:** Type A aortic dissection, Malperfusion syndrome, TEVAR, Hybrid surgery

## Abstract

**Objective:**

Acute aortic dissection type A is among the most lethal surgical emergencies. Patients may suffer from occlusion of the aorta or its branches causing end-organ malperfusion complicating the diagnosis and worsening the prognosis. Paraplegia is a rare manifestation that affects less than 5% of patients. If type A aortic dissection and occlusion of the downstream thoraco-abdominal aorta occur simultaneously and require acute treatment, a medical dilemma occurs; what should be treated first?

**Case report:**

We describe a case with an extensive acute type A aortic dissection with signs of consciousness and severe malperfusion syndrome.

**Results:**

The treatment was successfully performed within a hybrid surgery suite with simultaneous open surgery and endovascular repair techniques supported by cardiopulmonary bypass circulation.

**Conclusion:**

A hybrid operating room might offer the opportunity to simultaneously repair complicated aortic dissection with malperfusion syndrome, by open aortic surgery and endovascular techniques.

## Introduction

Acute aortic dissection type A is among the most lethal surgical emergencies. Stanford Type A dissection requires acute surgery, while Type B dissection is usually treated conservatively or with thoracic endovascular aortic repair (TEVAR) in case of rupture or malperfusion syndrome. In both A and B acute aortic dissection some patients may suffer from occlusion of the aorta or its branches causing end-organ malperfusion complicating the diagnosis and worsening the prognosis. Paraplegia is a rare manifestation that affects less than 5% of patients. If type A aortic dissection and occlusion of the downstream abdominal aorta occur simultaneously and require acute treatment, a medical dilemma occurs; what should be treated first?

We describe a case with acute Stanford classification type A aortic dissection with a propagation into the pelvic vessels, debuting with clinical signs of paraplegia and limb ischemia. The treatment was performed in our hybrid surgery suite with combined open surgery and endovascular repair techniques supported by cardiopulmonary bypass circulation (CPB).

## Clinical case

**Fig. 1:**
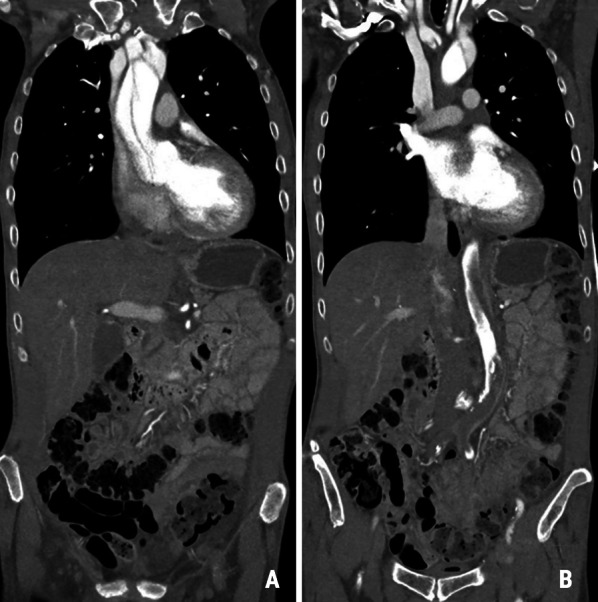
Aortic dissection type A (**A**) with subtotal occlusion of the descending and abdominal aorta (**B**) causing extensive distal organ malperfusion with subtotal occlusion of pelvic vessels

A 66-year-old man with known chronic obstructive pulmonary disease and multiple spine surgeries was admitted to the local hospital with acute paraplegia, critical limb ischemia; no palpable pulses, no capillary filling, white color and cold legs, back pain, bradycardia and loss of consciousness. Echocardiography suspected pericardial fluid and CT showed aortic Stanford type A dissection with extension into the neck vessels, the descending aorta and with occlusion of spinal arteries, kidney arteries and both pelvic vessels (Fig. 1A and B). After air ambulance transfer and admission to our regional cardiac surgery center three hours after onset of symptoms, the patient underwent emergency surgery in a hybrid operating room after interdisciplinary planning between the cardiothoracic surgeons and interventional radiologists. At admission to our hospital the patient was still spontaneously breathing with a moderate metabolic acidosis as pH, BE and S-lactate was 7.3, − 0.4, and 1.1 mmol/l, respectively.

**Fig. 2:**
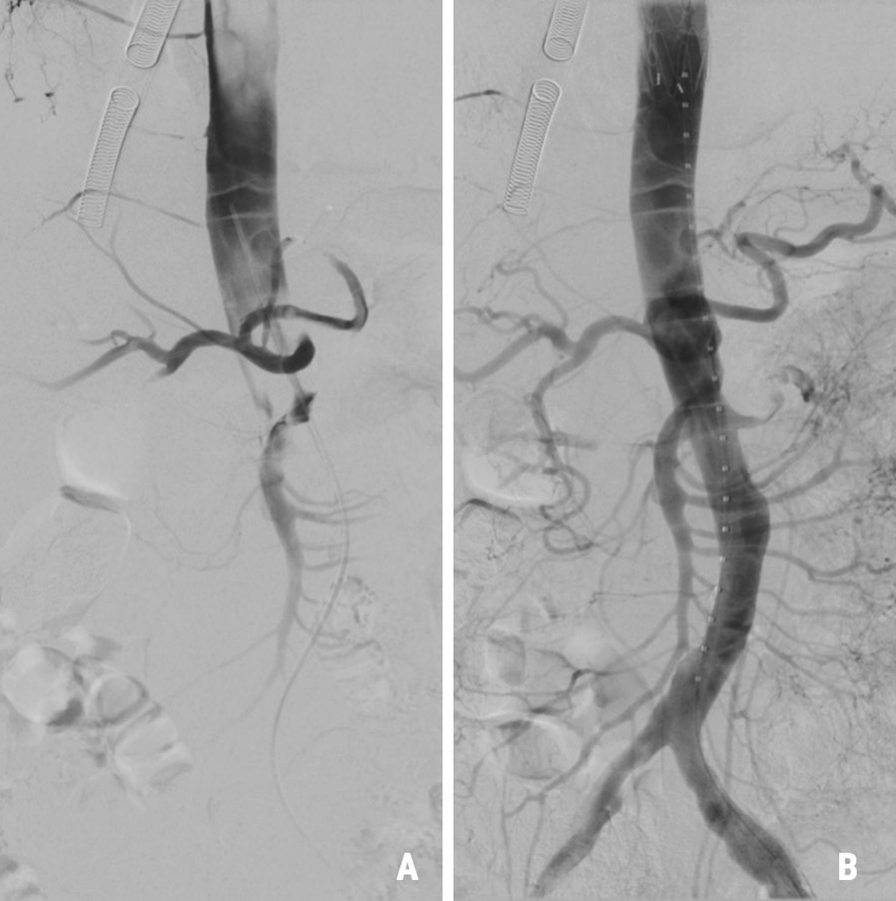
**A** Antegrade angiography showing improved perfusion in the descending aorta, but remaining occlusion in the abdominal aorta. **B** Stent graft implantation in the descending aorta with the subsequent improvement of the abdominal aortic circulation

Under general anesthesia and following sternotomy, direct aortic cannulation with EOPA 22F cannula (Medtronic, Minneapolis, USA) was performed into the thru lumen guided by epiaortic ultrasound (VeriQ-C ®, Medistim, Oslo, Norway). CPB with deep hypothermia was established and the patient was cooled to 20 °C. During cooling, intraoperative angiography was performed through a sideline of the aortic cannula showing widespread dissection with remaining occlusion of the downstream abdominal aorta and run-off vessels (Fig. 2A). A Bolton Relay 34/200 mm stent graft (Bolton Medical, Florida, USA) was implanted upstream into the descending aorta via the left common femoral artery, which reestablished circulation to the lower extremities and the spinal cord (Fig. 2B). The covered part of the stent graft were placed close to the left subclavian artery, and only the bare string ended in zone 2, hence the left subclavian artery was not occluded. At 20 °C esophageal temperature the ascending aorta was opened and bilateral antegrade cerebral perfusion was commenced by direct cannulation into the aortic arch vessels. With luminal view of the aortic arch, the ascending aorta was replaced with a Vascutek Gelweave Ante-Flo 28/8 tube graft (Vascutek Terumo, Florida, USA).

**Fig. 3:**
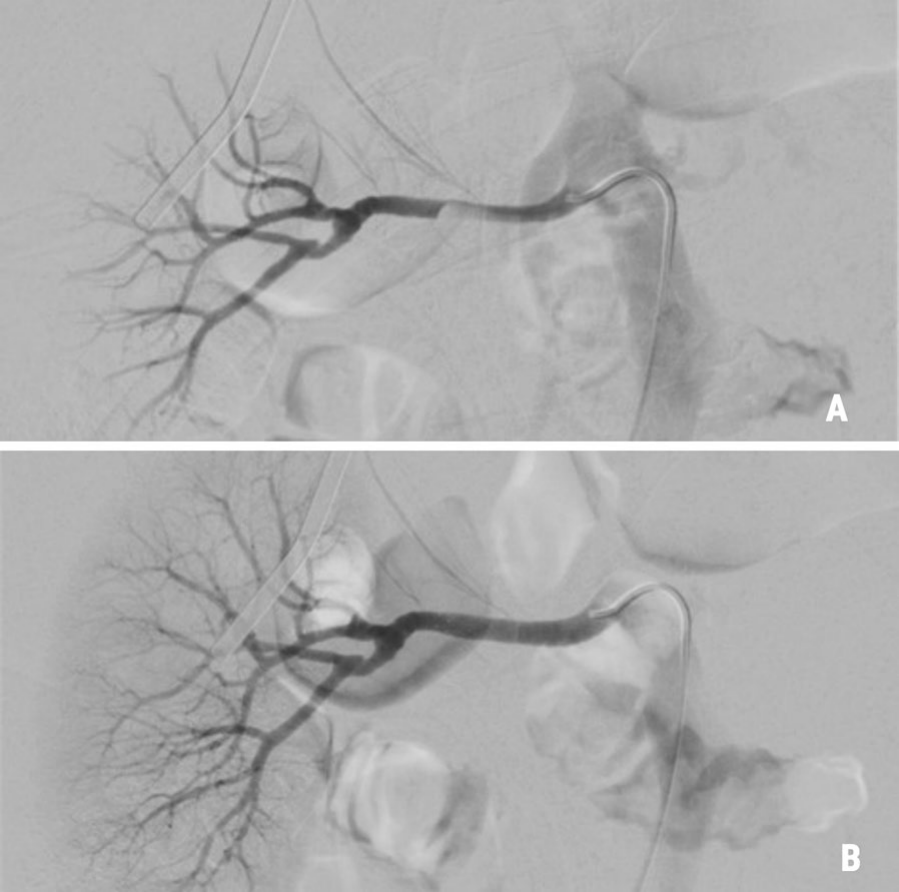
Stenosis (**A**) of the right renal artery treated with a stent assuring good perfusion to the right kidney (**B**)

**Fig. 4:**
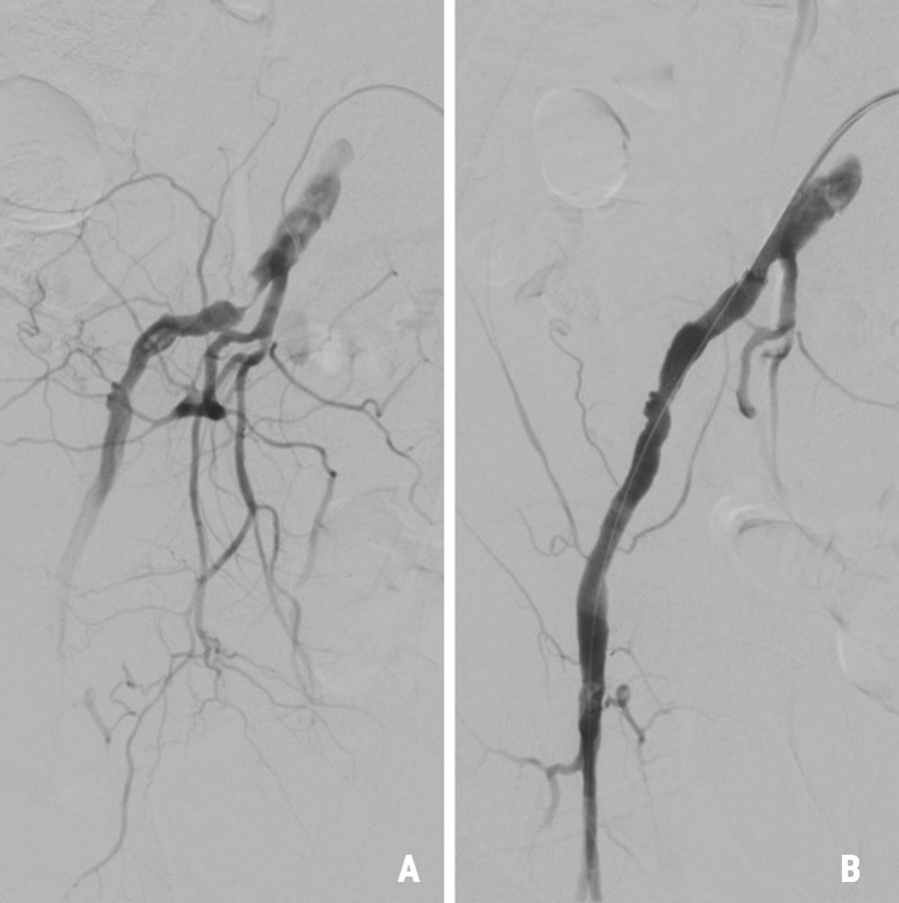
Occlusion of the right iliac artery before (**A**) and after stenting (**B**)

Following the distal anastomosis, downstream blood flow and systemic circulation was resumed via the sidearm of the prosthesis. During rewarming, repeated angiography showed renal artery stenosis limiting perfusion of the right kidney and dissection with subtotal occlusion of the iliac vessel on the right side. The endovascular procedure was terminated with stenting of both the right renal artery with a Everflex 7/40 mm (Medtronic, Minneapolis, USA) and the right external iliac artery with a Protege 10/40 mm (Medtronic, Minneapolis, USA), each with good angiographic results confirming patent distal run-off (Figs. 3A, B and 4A,  B).

Following surgery, ventilatory support could be ended on the first postoperative day. The further postoperative course was uneventful without new onset of ischemic signs and few hours after waking up he gradually regained lower limb function. Following discharge to his local hospital, the patient underwent an extended rehabilitation program and could walk about after 3 months.

## Discussion

Acute aortic dissection is a potentially catastrophic condition, and patients presenting preoperatively with malperfusion syndromes have significantly increased risk of mortality and postoperative complications [1–4]. The dilemma concerning what to treat first may frequently occur with risking a rupture of the ascending aorta or risking a disastrous irreversible end-organ ischemia. Several authors report of successful treatment of a staged approach [1, 5, 6] performing percutaneous interventional procedures with fenestration and distal stent-grafting of the descending aorta first, with a subsequent delayed trans-sternal aortic repair in deep hypothermia. Others advocate for an immediate proximal aortic repair [7–9]. It is important to keep in mind, even though delayed central surgery may be a tempting alternative in certain subgroups, that mortality rates for conservatively or delayed treatment of type A aortic dissection are substantial and directly related to time from onset [10].

The disease debuts with various symptoms and different approaches might be useful, depending on the patient's clinical situation, CT scan, blood tests and cardiac deterioration due to hemopericardium or aortic leak as assessed by echocardiography. While observation of pericardial effusion and risk of tamponade indicates that surgical repair cannot be delayed, serum lactate has been suggested as a useful indicator of ischemia [6], hence the degree of metabolic disturbance might advocate postponing repair of malperfusion issues. The need for limb revascularization is, regardless, a marker for more extensive dissection, and these patients are more likely to have mesenteric ischemia [11].

**Fig. 5:**
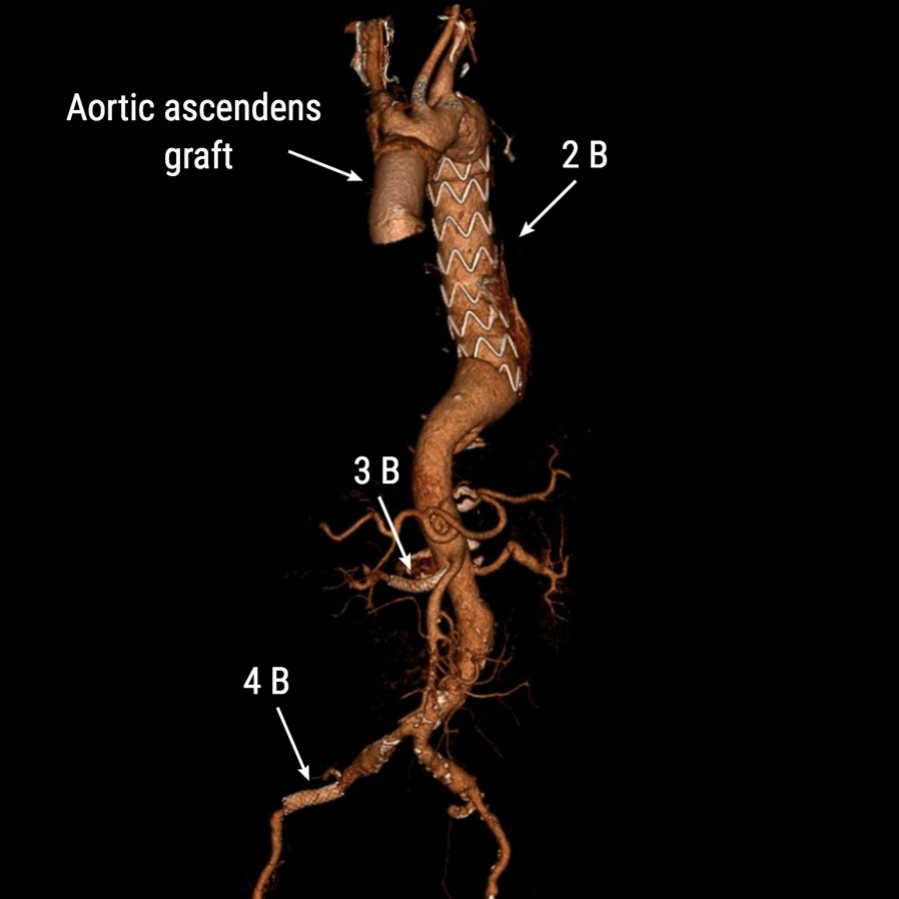
Schematic drawing of the complete hybrid procedure of open surgery with supracoronary tubegraft in the ascending aorta, thoracic aortic stent-grafting and stents in the right renal artery and right external iliac artery

Our patient had an extensive type A aortic dissection with signs of consciousness and severe malperfusion syndrome. As the lacto-acidosis was still moderate, the complete surgical treatment was performed in a hybrid suite, located in the regional cardiothoracic surgery center. After an immediate sternotomy with establishment of cardiopulmonary bypass and deep hypothermia, the ruptured ascending aorta was secured. Without delay and during cooling of the patient, percutaneous stent-grafting of the descending aorta was done to limit the time of malperfusion and avoiding severe end-organ ischemia. Subsequently, ascending aortic repair was completed and following repeated angiography residual right renal and right iliac artery stenosis could be stented (Fig. 5).

## Conclusion

Acute aortic dissection type A complicated by extensive malperfusion syndrome including the spinal cord, kidneys and lower limb can be treated successfully in a hybrid operating room with simultaneous open aortic surgery and endovascular aortic and distal artery repair techniques.

## Abbreviations

TEVAR: Thoracic endovascular aortic repair
CPB: Cardiopulmonary bypass circulation (CPB)
CT: Computed tomography
